# Anatomical Study of the Zygomaticotemporal Branch Inside the Orbit

**DOI:** 10.7759/cureus.1727

**Published:** 2017-09-29

**Authors:** Joe Iwanaga, Charlotte Wilson, Koichi Watanabe, Rod J Oskouian, R. Shane Tubbs

**Affiliations:** 1 Seattle Science Foundation; 2 Department of Anatomy, Kurume University School of Medicine; 3 Neurosurgery, Complex Spine, Swedish Neuroscience Institute; 4 Neurosurgery, Seattle Science Foundation

**Keywords:** orbit, zygomatic bone, maxillary nerves, surgery, orbital fractures

## Abstract

The location of the opening of the zygomaticotemporal branch (ZTb) of the zygomatic nerve inside the orbit (ZTF_IN_) has significant surgical implications. This study was conducted to locate the ZTF_IN_ and investigate the variations of the ZTb inside the orbit. A total of 20 sides from 10 fresh frozen cadaveric Caucasian heads were used in this study. The vertical distance between the inferior margin of the orbit and ZTF_IN _(V-ZTF_IN_), the horizontal distance between the lateral margin of the orbit and ZTF_IN_ (H-ZTF_IN_), and the diameter of the ZTF_IN_ (D-ZTF_IN_) were measured. The patterns of the ZTb inside the orbit were classified into five different groups: both ZTb and LN innervating the lacrimal gland independently (Group A), both ZTb and LN innervating the lacrimal gland with a communicating branch (Group B), ZTb joining the LN without a branch to the lacrimal gland (Group C), the ZTb going outside the orbit through ZTF_IN_ without a branch to the lacrimal gland nor LN (Group D), and absence of the ZTb (Group E). The D-ZTF_IN_ V-ZTF_IN_ H-ZTF_IN_ ranged from 0.2 to 1.1 mm, 6.6 to 21.5 mm, 2.0 to 11.3 mm, respectively. The number of sides in Groups A, B, C, D, and E were 13 sides (65%), three sides (15%), none (0%), two sides (10%), and two sides (10%), respectively. Such anatomical knowledge might reduce complications following surgery in and around the ZTb.

## Introduction

The zygomaticotemporal branch (ZTb) of the zygomatic nerve, which is a branch of the maxillary division of the trigeminal nerve, runs along the lateral wall of the orbit. It passes through the zygomaticotemporal foramen (ZTF) and then travels in the temporal fossa [1]. It has a communicating branch with the auriculotemporal and facial nerves [2-3]. Inside the orbit, many anatomical depictions have shown a communicating branch between the ZTb and the lacrimal nerve (LN) [4]. The ZTb carries sensory fibers to the temporal region and receives parasympathetic fibers from the pterygopalatine ganglion that are destined to innervate the lacrimal gland directly or indirectly through the LN. According to Scott et al., however, 60.6% of the cadaveric heads did not have any communicating branches between the ZTb and LN [5]. In addition, the ZTb has variations in its course inside the orbit. Most studies of the ZTb have investigated the course in the temporal fossa in terms of surgery of the temporal fossa and temporal approaches [2,4,6]. From the surgical point of view, the location of the opening of the ZTb inside the orbit (ZTF_IN_) is also important and the existence of the communicating branch might impact the surgical procedure. Thus, a more detailed anatomy of the ZTb inside the orbit is necessary to better understand and reduce complications. Therefore, this study was conducted to locate the ZTF_IN_ and investigate the variation of the ZTb inside the orbit.

## Materials and methods

**Figure 1:**
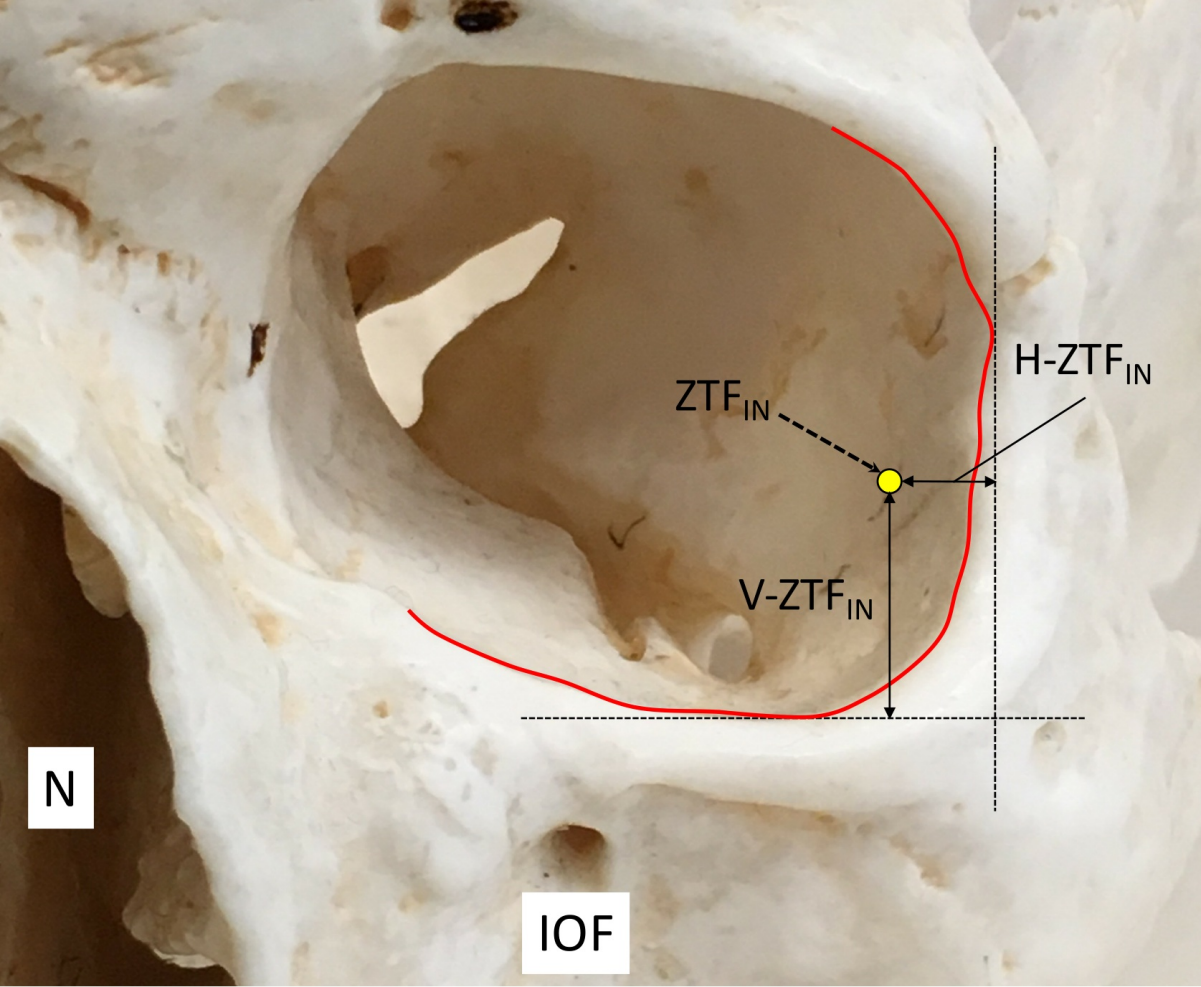
Measurement of the horizontal and vertical distances of the ZTFIN. Note the lateral and inferior margin of the orbit is colored in red (left orbit). N; nasal cavity, IOF; infraorbital foramen, ZTF_IN; _the opening of the ZTb inside the orbit, H-ZTF_IN_; horizontal distance between the lateral margin of the orbit and ZTF_IN_, V-ZTF_IN_; the vertical distance between the inferior margin of the orbit and ZTF_IN_.

A total of 20 orbits from 10 fresh frozen cadaveric Caucasian heads were used in this study. The specimens were derived from two males and eight females and the age at death ranged from 63 to 90 years (mean age: 78.0±7.9 years old). In the supine position, a skin incision was made along the inferior to lateral margin of the orbit. First, the periosteum inside the orbit was elevated superiorly and medially to identify the ZTF_IN_. Next, the vertical distance between the inferior margin of the orbit and ZTF_IN_ (V-ZTF_IN_), the horizontal distance between the lateral margin of the orbit and ZTFIN (H-ZTF_IN_), and the diameter of the ZTF_IN_ (D-ZTF_IN_) were measured. A horizontal reference line was used parallel to the line between the right and left pupils and a vertical line was made vertical to the horizontal reference line (Figure 1). Finally, the patterns of the ZTb inside the orbit were investigated and classified into five different groups; both ZTb and LN innervating the lacrimal gland independently (Group A), both ZTb and LN innervating the lacrimal gland with a communicating branch (Group B), ZTb joining the LN without a branch to the lacrimal gland (Group C), the ZTb going outside the orbit through the ZTF_IN_ without a branch to the lacrimal gland or LN (Group D), and absence of the ZTb (Group E).

Two clinical anatomists performed all dissections and measurements. Dissections were carried out under a surgical microscope (OPMI CS NC31, Carl Zeiss, Oberkochen, Germany), and measurements were made with a microcaliper (Mitutoyo, Kanagawa, Japan) with a resolution of 0.01 mm and an accuracy value of ±0.025 mm. The measurements were performed three times by each observer (for a total of six times for each measurement) and then averaged. The present study protocol did not require approval by the ethics committees in our institutions, and work was performed in accordance with the requirements of the Declaration of Helsinki (64th WMA General Assembly, Fortaleza, Brazil, October 2013).

## Results

The total number of ZTF_IN_ identified was 18. The number of ZTF_IN_ on each side ranged from 0 to 1; none on two sides (10%), and one on 18 sides (90%). The D-ZTF_IN_ ranged from 0.2 to 1.1 mm with a mean of 0.7±0.2 mm (0.6±0.2 mm on the right sides and 0.7±0.2 mm on the left sides). The V-ZTF_IN_ ranged from 6.6 to 21.5 mm with a mean of 15.0±4.0 mm (14.0±4.3 mm on the right sides and 16.1±3.6 mm on the left sides). The H-ZTF_IN_ ranged from 2.0 to 11.3 mm with a mean of 6.0±2.6 mm (5.9±2.8 mm on the right sides and 6.2±2.5 mm on the left sides) (Table 1). There were no statistically significant differences between the right and the left sides regarding D-ZTF_IN_, V-ZTF_IN_, and H-ZTF_IN_ (p > 0.05).

**Table 1:** Diameter and distance of ZTFIN ZTF_IN_;the opening of the ZTb inside the orbit, D-ZTF_IN_; diameter of the ZTF_IN_, H-ZTF_IN_; horizontal distance between the lateral margin of the orbit and ZTF_IN_, V-ZTF_IN_; vertical distance between the inferior margin of the orbit and ZTF_IN _

	Range (mm)	Mean (mm)
D-ZTF_IN_	0.2 – 1.1	0.7 ± 0.2
V-ZTF_IN_	6.6 – 21.5	15.0 ± 4.0
H-ZTF_IN_	2.0 – 11.3	6.0 ± 2.6

**Figure 2:**
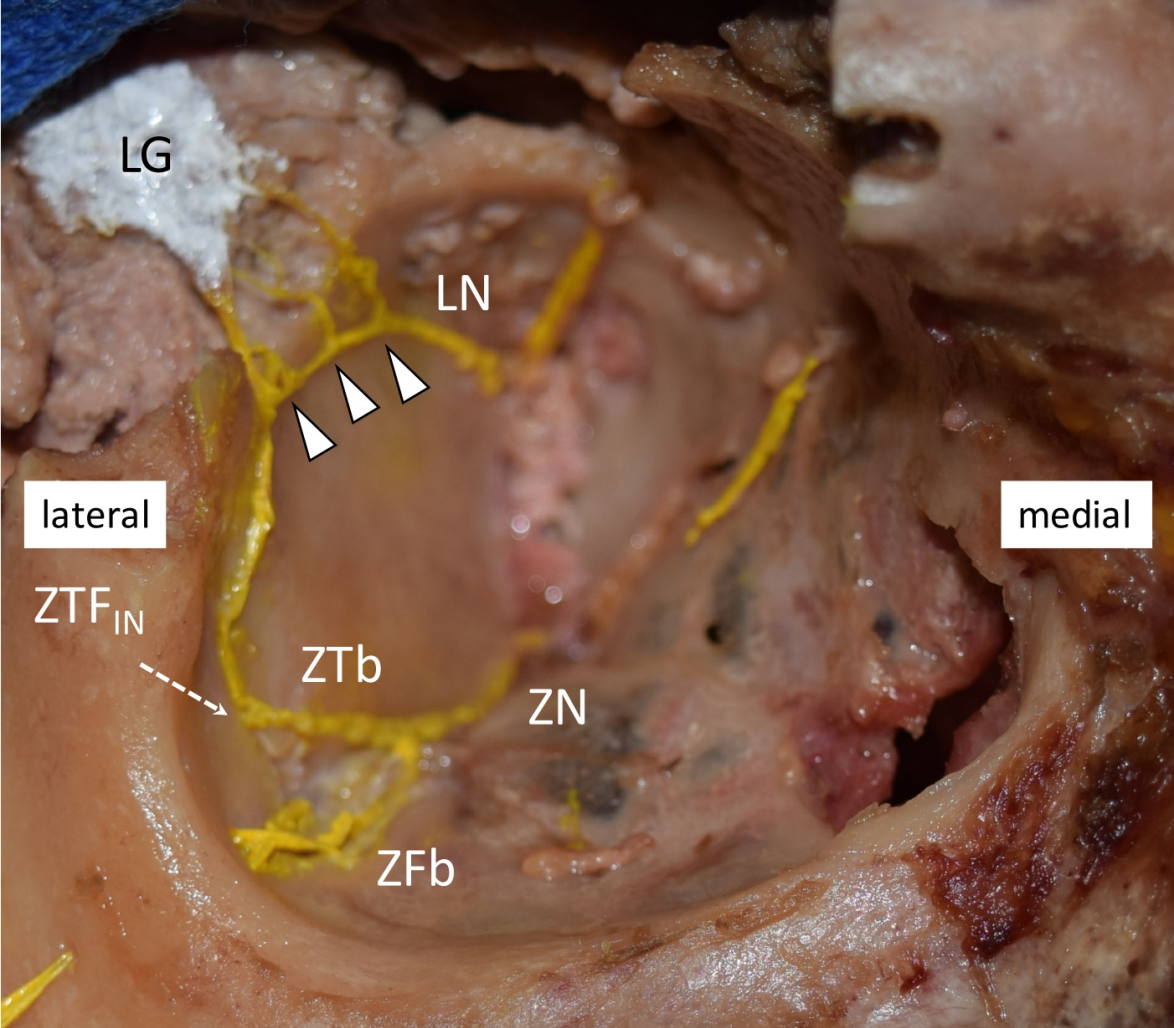
Dissection of Group B on the right side. Both the ZTb and the LN innervated LG and had a communicating branch (arrowheads). LG; lacrimal gland, LN; lacrimal nerve, ZFb; zygomaticofacial branch, ZN; zygomatic nerve, ZTb; zygomaticotemporal branch, ZTF_IN; _the opening of the ZTb inside the orbit.

For the patterns of the ZTb inside the orbit, 13 sides (65%) were classified as Group A, three sides (15%) as Group B (Figure 2), none (0%) as Group C, two sides (10%) as Group D, and two sides (10%) as Group E (Table 2). Of these, two sides in Group A had no ZTF_IN_ but ZTb directly innervated the lacrimal gland without a communicating branch to the LN. Additionally, the other two sides in Group E had an unusual branch, which arose from the LN and passed through the ZTF_IN_ after giving rise to a branch to the LN.

**Table 2:** Running patterns of the ZTb (zygomaticotemporal branch) inside the orbit Group A; both ZTb and LN innervating the lacrimal gland independently, Group B; both ZTb and LN innervating the lacrimal gland with a communicating branch, Group C; ZTb joining the LN without a branch to the lacrimal gland, Group D; the ZTb going outside the orbit through the ZTF_IN_ without a branch to the lacrimal gland or LN, Group E; absence of the ZTb

	n	%
A	13	65
B	3	15
C	0	0
D	2	10
E	2	10

## Discussion

Previous anatomical studies of the ZTF have revealed morphological features of the foramina on the lateral surface of the zygomatic bone [7-8]. Kim et al. investigated the ZTF using micro-computed tomography (micro-CT) and found that the bony canal often bifurcates inside the zygomatic bone [9]. However, the bony canals identified in dry skulls or on CT could include not only the nerves but also vessels. In the present study, only the nerves passing through the foramina were focused on and dissected using a surgical microscope.

Hwang et al. used the zygomaticofrontal suture and zygomatic arch as a reference point to investigate the ZTb [10]. This study showed that the D-ZTF_IN_, V-ZTF_IN_, and H-ZTFIN ranged from 0.2 mm to 1.1 mm with a mean of 0.7±0.2 mm, 6.6 mm to 21.5 mm with a mean of 15.0±4.0 mm, and 2.0 mm to 11.3 mm with a mean of 6.0±2.6 mm, respectively. This shows that the ZTF_IN_ could be identified near the lateral margin of the orbit but almost in the middle of the orbit in terms of height.

It has been traditionally taught that the lacrimal nerve does not carry parasympathetic fibers to the lacrimal gland but the ZTb does, so that the communicating branch between the ZTb and LN exists in most cases. However, recently, Scott et al. reported that a communicating branch was found in less than 40% of the cases [5]. Compared to Scott et al.’s study, Group A was 65% in our study (60.6% in Scott’s study), Group B was 15 % in our study (36.4% in Scott’s study), and Group C was 0% in our study (3% in Scott’s study). Neither Group D nor Group E was classified in Scott et al.’s study. Group D was 10% and had no innervation to the lacrimal gland. Group E was 10% without any ZTb but the lacrimal nerve passed through the ZTF_IN_. The pathways of the parasympathetic fibers described in textbooks do not explain the innervation in Groups D and E because, in these groups, the lacrimal gland only received its nerve supply from the LN. In such cases, the LN should carry the parasympathetic fibers but immunohistochemistry is necessary to prove this.

The human zygomatic bone has one to three ossification centers. These appear in the eighth week of gestation and fuse at about the 22^nd^ week [11]. The embryological origin of the variations of the ZTF may be due to the variations of its ossification centers and this might result in various numbers of foramina. Based on anatomical studies of the mandible, Iwanaga et al. speculated that the mental foramen is formed by the addition of bone around the nerve bundles proximal to the branching point, and accessory mental foramina are formed by bone addition peripheral to the branching point [12-13]. This could be applied to the formation of multiple ZTF_IN_. If the bone addition occurs around the ZTb bundles before giving rise to branches, theoretically, only one ZTF_IN_ would be formed. However, if the bone addition occurs after giving off branches, it might form an additional zygomaticotemporal foramen.

## Conclusions

Our results revealed a variation in the distribution of the ZTb. Occasionally, the ZTb did not innervate the lacrimal gland where its only innervation was via the LN. An immunohistochemical examination is now required to identify parasympathetic fibers inside the ZTb and LN in order to better understand the innervation of the lacrimal gland.
